# A friction compensation approach of a 6 axis hybrid robot by considering joint inertia change

**DOI:** 10.1016/j.isci.2025.112173

**Published:** 2025-03-06

**Authors:** Qi Liu, Sitong Shen, Yue Ma, Bin Li

**Affiliations:** 1 Tianjin Key Laboratory for Advanced Mechatronic System Design and Intelligent Control, School of Mechanical Engineering, Tianjin University of Technology, Tianjin 300384, China; 2 National Demonstration Center for Experimental Mechanical and Electrical Engineering Education, Tianjin University of Technology, Tianjin 300384, China

**Keywords:** Mechanical modeling, Mechanical engineering, Robotics

## Abstract

The feedforward compensation based on friction model is an effective way to reduce the influence of friction on mechanical system. This paper presents an approach for friction compensation by considering the inertia change of the actuated joints of a 6-axis hybrid robot named TriMule. First, the tracking errors of each actuated joint of the parallel mechanism at high and low inertia configurations are displayed. Then, a compensation approach considering inertia change is developed by introducing the inertia term of the joint driving force into the traditional Stribeck model as thrust. Furthermore, the improved approach based on radial basis function interpolation without using the dynamic model is proposed, which has a simple calculation expression and acceptable compensation effect. Experiment results on a prototype machine show that compared to otherwise similar compensation method not considering joint inertia change, the tracking accuracy can be improved up to 25.73% at high inertia configurations.

## Highlights

• Friction contribution proportion between the parallel mechanism and the wrist is revealed
• Tracking errors of each parallel actuated joint at different configurations are displayed
• A compensation approach considering inertia change is developed
• Radial basis function interpolation is used to simplify the calculation

## Graphical abstract

**Figure:**
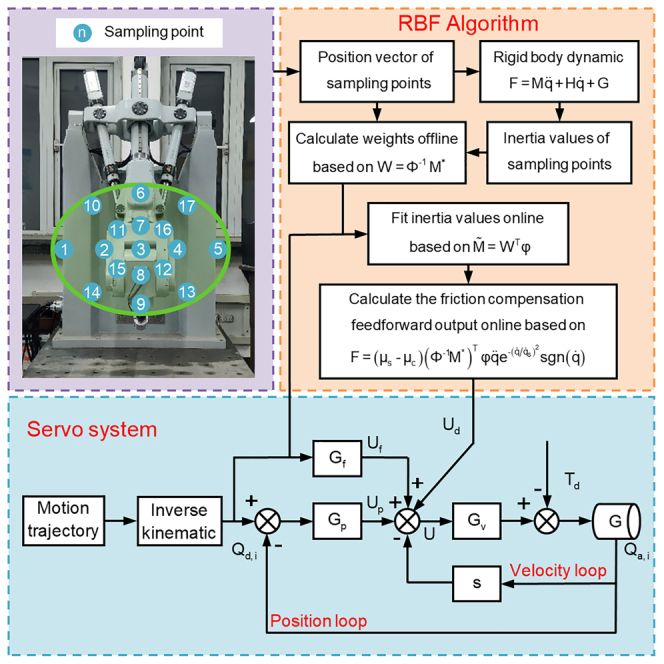

## Introduction

Friction is a complex nonlinear phenomenon that exists in almost all mechanical systems, which leads to many problems, such as limit cycle oscillation and slip motion at low velocity and commutation.^1^^,^^2^^,^^3^ Due to the complex nonlinear structural characteristics of robots, it is more difficult to analyze trajectory errors caused by friction, which reduces the control accuracy and the stable level of the motor drive system.^4^^,^^5^ Therefore, friction compensation is an important issue in the design of robot feedforward controllers.

Friction compensation divides roughly into two categories: indirect methods and direct method.^5^ For the methods belonging to the first category, the anti-interference ability of the system is improved by tuning feedback controller parameters to reduce the influence of friction disturbance.^6^^,^^7^^,^^8^^,^^9^ However, the improvement effect is limited when the fast response performance of the system is usually needs to be taken into account. The direct methods in dealing with friction compensation can also be roughly classified into two categories: model-based methods and data-driven methods. In model-based compensation, the friction disturbance is calculated by designing the algorithm describing the frictional contact process.^10^^,^^11^^,^^12^ The latter uses neural network and other methods to construct a prediction model that can predict friction disturbance based on data training.^13^^,^^14^^,^^15^^,^^16^ Considering the complex structure and training process of neural networks, model-based methods are more suitable for simplifying algorithms and implanting them into controllers.^17^ Therefore, tremendous efforts have been made toward the improvement of the friction model accuracy and the compensation effect.^18^ Studies have shown that friction phenomenon can be divided into four stages, comprising static, boundary lubrication, partial fluid lubrication and full fluid lubrication friction, which are related to the relative motion velocity between the contact surfaces of two objects.^4^ A large number of friction models have been proposed to describe these characteristics in depth. Friction models can be divided into static friction models and dynamic friction models according to whether the differential equation is used to describe them. The static friction model is expressed as the velocity function, including the Karnopp model^19^ and Stribeck model,^20^ etc. The dynamic friction model is expressed as a function of velocity and displacement, including the Dahl model,^21^ LuGre model,^22^ etc. In addition, the effects of temperature, force, joint angle, joint velocity, joint acceleration, and lubricant performance have been focused on by many scholars in order to further improve the accuracy.^23^^,^^24^

In the field of friction compensation aimed at improving accuracy (model-based), one method is to design compensators utilizing the joint position and its first and second derivatives. By fitting the tracking error caused by nonlinear friction at different positions of the double-gimbal control moment gyro (DGCMG), Han et al.^25^ proposed an improved Coulomb-Viscous model which combines the change of angular position, the tracking error of angular velocity introduced by the nonlinear friction of harmonic drive is restrained effectively. Bui et al.^26^ proposed a nonlinear friction model that includes the typical Coulomb-Viscous model and a nonlinear sinusoidal friction term for describing the lead screw property to compensate for the irregular friction disturbance caused by the eccentricity of the screw when the lubrication is insufficient. Xi et al.^27^ proposed a two-stage tracking error-based static friction compensation scheme to compensate for static friction during the transition from the pre-sliding regime to the sliding regime. The model consists of two phases, the first is a function of tracking error and the second is a function of tracking error and velocity.

However, this method may not achieve ideal compensation effects in some complex mechanical systems. Though some works designed compensators by considering the connection between the driving force and friction, the parameters in these models are generally constants for simplified calculations. Bittencourt et al.^28^ established a very comprehensive multi-scale model to describe the interplay between speed, load, temperature, and wear. However, the driving force component as a parameter of the model was calculated under the condition that the inertial torque was zero, leading to the applied torque only contains gravity-induced torque and friction.

As a robot for processing large-scale thin-walled structural parts, the TriMule robot boasts a wide range of workspaces.^29^ The significant change in inertia from the central to the edge configuration poses a challenge for friction compensation. The configuration change of the robot leads to variations in the inertia of each actuated joint, resulting in difference in friction disturbances. How to achieve friction compensation across the entire workspace on the basis of considering the aforementioned issues is rarely addressed in existing studies. The key problem in precisely implementing friction compensation for the TriMule robot lies in how to use a friction model to reflect the impact of the inertia variation with configuration changes on friction disturbances. Therefore, by analyzing the relationship between friction and the inertia of parallel mechanism actuated joints of TriMule robot, a friction compensation approach considering the inertia change with configurations is proposed in this paper, aiming to achieve accurate compensation for friction disturbance across the entire workspace.

Having reviewed the methods for friction compensation methods in the Introduction, the proportion of the tracking error of each actuated joint caused by friction mapped to the circular trajectory is analyzed, and the law of the friction disturbance affected by the inertia change of each actuated joint of parallel mechanism is revealed. By taking account of the inertia change, an approach for compensating friction arising from robot configuration change is presented. A dynamic based compensation algorithm is proposed first by introducing the inertia term of the driving force into the traditional stribeck model as thrust. Then, a novel approach for fitting the inertia variation in the friction model using radial basis function (RBF) interpolation is developed for the compensation of friction disturbance. In the results section, experiments are carried out on a prototype machine to verify the effectiveness of the proposed approach before conclusions and limitations of the study are drawn in the discussion section.

### Robot system description and friction analysis

In this section, the contribution proportion of the friction effect between the parallel mechanism and the serial wrist will be revealed by analyzing the tracking errors at quadrant crossing positions on the circular trajectory after a brief introduction to the structure of the TriMule robot. This is followed by the investigation into the influence of inertia change on the magnitude of friction disturbance.

#### System description

**Figure 1:**
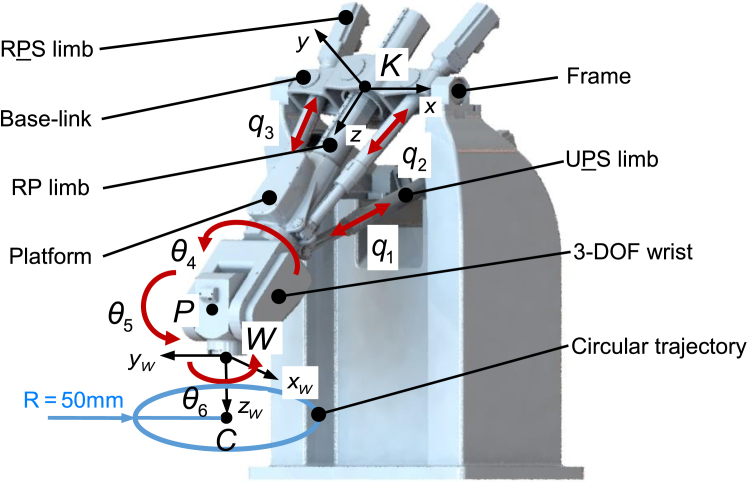
TriMule robot CAD model

Figure 1 shows the CAD view of the TriMule hybrid robot, which consists of a 3-DOF 1T2R (T—Translation, R—Rotation) parallel mechanism and a 3-DOF wrist. The 1T2R parallel mechanism consists of the base-link, one RP limb, one UPS limb, and two RPS limbs, the ends of these limbs are connected to the platform. Here, R, P, U and S represent revolute, prismatic, universal, and spherical joints respectively, and the underlined P represents an actuated prismatic joint.

For convenience, the two RPS actuated limbs, the UPS actuated limb and the RP passive limb are denoted as limb i (i=1,2,3,4), the length of each limb is denoted by $q_{i}\left(i=1,2,3,4\right)$. The three revolute joints of the wrist are denoted as joint j(j=4,5,6), the angle of each joint are denoted by, and *P* is set as the intersection point of the three orthogonal axis of the wrist. Κ−xyz is the reference coordinate system with the *x* axis being the axial line of the R joint connecting the base-link and the frame, and the *y* axis being parallel to the plane stretched by the frame of three parallel actuated limbs. $W-x_{w}y_{w}z_{w}$ is the workpiece coordinate system fixed above the workbench and serves as the reference for controlling the movement of the robot. The $x_{w}$ axis is parallel to and in the same direction as the x axis of the Κ−xyz system, the $z_{w}$ axis points vertically down to the workbench, and the $y_{w}$ axis are determined according to the right hand rule of Cartesian coordinate system.

#### Friction analysis

Figure 1 also shows the circular trajectory with a radius of 50 mm in the $x_{w}-y_{w}$ plane of the $W-x_{w}y_{w}z_{w}$ coordinate system. The tool axis keeps straight down when the robot executes the trajectory. Here, joint i (i=1,2,3) is the actuated joint of the parallel mechanism, and joint i (i=4,5,6) is that of the wrist.

**Figure 2:**
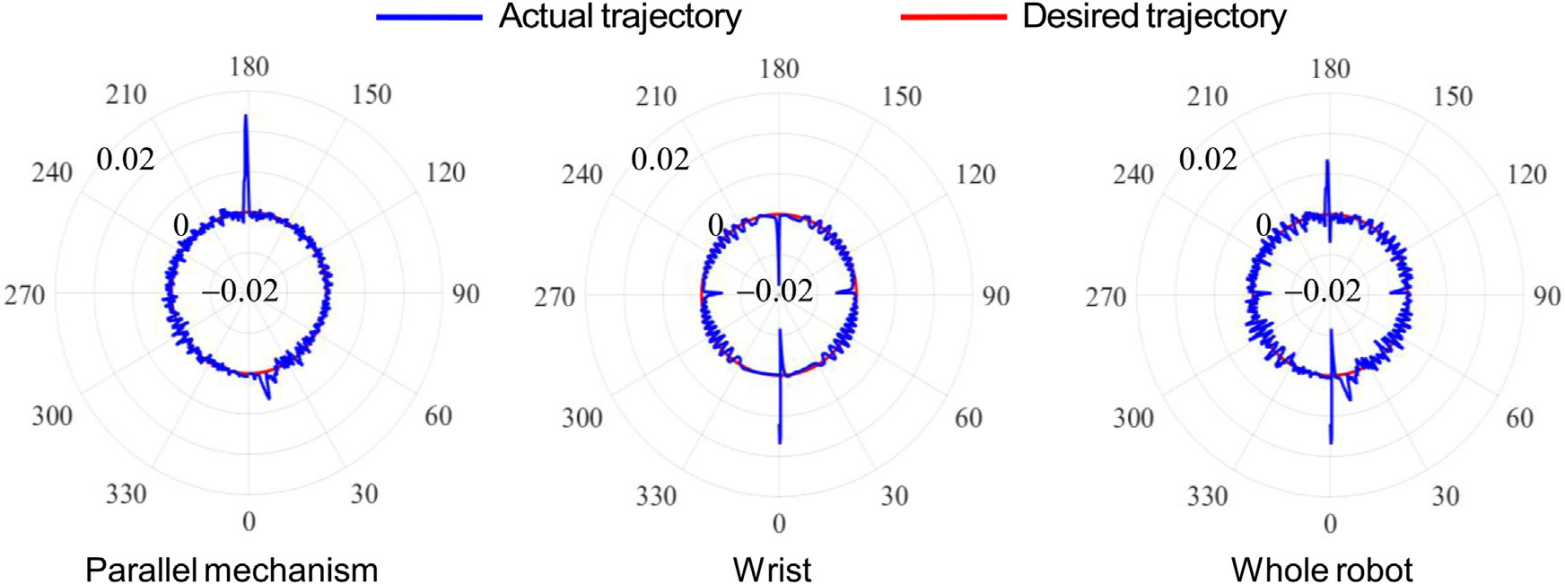
The polar coordinates contour of the tracking errors (mm) contributed by the parallel mechanism, the wrist, and the whole robot

**Figure 3:**
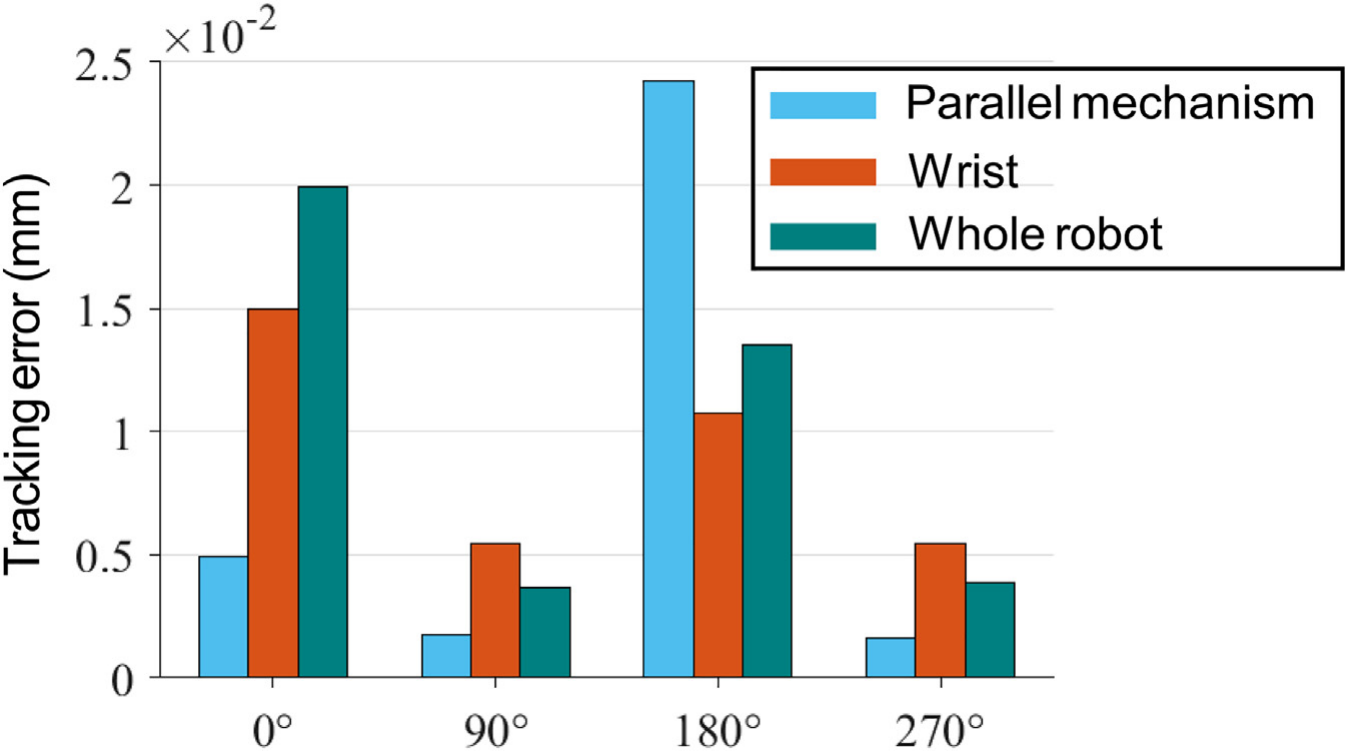
The proportion of the tracking errors contributed by parallel mechanism and the wrist in the mapping of that contributed by the whole robot at quadrant crossing positions

Figure 2 shows the polar coordinates contour of the tracking errors contributed by the parallel mechanism (i=1,2,3), the wrist (i=4,5,6), and the whole robot (i=1,2,..,6) respectively, which are mapped to the circular trajectory. The display range is limited to between 49.98 mm and 50.03 mm. As shown in Figure 2 (Parallel mechanism), the prominent errors appear in the $0^{o}$ and $180^{o}$ neighborhood when the friction contributed by the parallel mechanism (i=1,2,3) is only considered. As shown in Figure 2 (Wrist), the prominent errors appear in all quadrants when the friction contributed by the wrist (i=4,5,6) is considered. Simultaneously taking into account the aforementioned friction factors, the errors are especially obvious in the $0^{o}$ and $180^{o}$ neighborhood, and slightly larger in the $90^{o}$ and $270^{o}$ neighborhood than that of other positions as shown in Figure 2 (Whole robot). Figure 3 shows the proportion of the tracking errors contributed by parallel mechanism and the wrist in the mapping of that contributed by the whole robot at the locations of $0^{o}$, $90^{o}$, $180^{o}$, and $270^{o}$. The errors contributed by the parallel mechanism in the $180^{o}$ neighborhood is the most prominent, and the errors caused by the wrist plays a leading role in the $0^{o}$, $90^{o}$, and $270^{o}$ neighborhood. It can be seen from the figures that the friction disturbance generated by the parallel mechanism and the wrist cannot be ignored.

**Figure 4:**
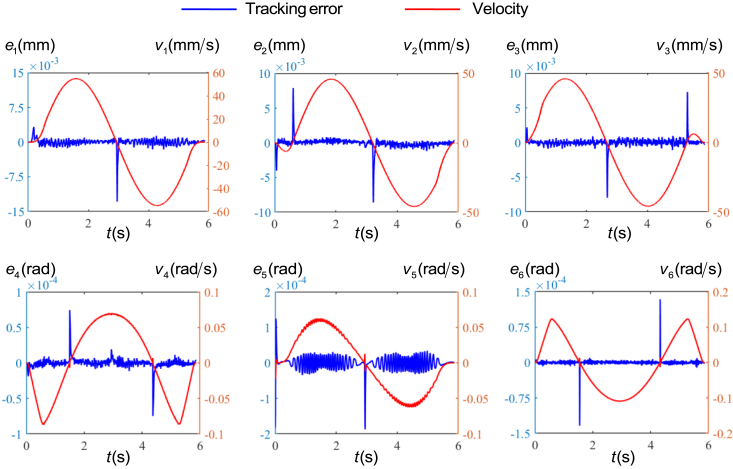
The tracking errors and velocity of each actuated joint with time for the circular path planned in the operation space

Figure 4 shows the tracking error $e_{i}$ and velocity $v_{i}$ of each actuated joint with time for the circular path planned in the operation space. It can be found that the tracking errors change suddenly at the start and commutation, and the direction of the tracking error mutation is consistent with the changing trend of the velocity. Among them, the peak error of the 1, 2, and 3 axis is less than 15μm, and the peak error of the 4, 5, and 6 axis is less than 2 × 10^−4^ rad.

It can be seen from Figure 4 that the static friction during the start-up and reversal stages causes a sudden change in tracking errors, while the inertial term in the driving force plays a dominant role in this stage. Inertial force consists of acceleration and inertia, yet most existing studies focus on the effect of acceleration changes on static friction, while assuming inertia as a constant value. The friction compensation function of Siemens 840D control system also eliminates sudden changes in tracking errors during startup or reversal by measuring the curve of tracking errors varying with acceleration.^30^

**Figure 5:**
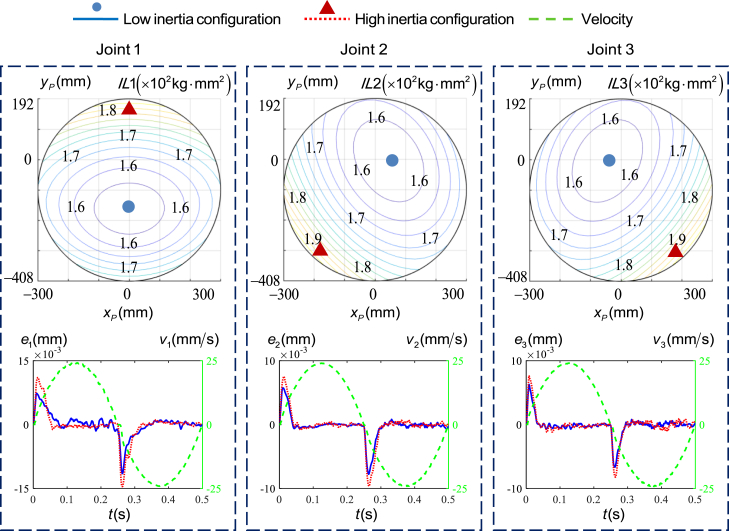
The inertia contour map and the tracking errors at the low and high inertia configurations of each actuated joint of the parallel mechanism in middle layer of the task workspace

However, the inertia of each actuated joint in the parallel mechanism of TriMule robot varies with the configuration, resulting in different tracking errors caused by static friction under the same acceleration and deceleration conditions, as shown in Figure 5, while that of the wrist can be regarded as approximately unchanged.^31^ The distribution of inertia was calculated in the central plane (x−y plane) of the task workspace of point *P* in Κ−xyz coordinate system through rigid dynamics of the parallel mechanism. Then the inertia contour map and the low and high inertia configurations can be drawn as shown in Figure 5. In order to explore the influence of the inertia change on the friction, different configurations of parallel mechanism are selected to test the changes of tracking errors caused by friction, at which each actuated joint performed a short reciprocating motion. Figure 5 also shows the tracking errors of each actuated joint at the low and high inertia configurations.

It can be seen from the figure that the tracking error caused by friction varies with the inertia or the configuration. The vector position of point *P* in Κ−xyz coordinate system and the peak values of tracking errors at low and high inertia configurations are listed in Table 1. Compared with moving at low inertia configurations, the tracking errors caused by friction of joint 1, 2, and 3 increase by 21.03%, 19.50%, and 19.08%, respectively, at the high inertia configurations. This confirms the argument that considering the inertia change helps significantly to improve the joint tracking accuracy, especially when the parallel mechanism operates at different configurations.

**Table 1:** The vector position of point *P* i**n**Κ−xyz coordinate system and the peak values of tracking errors at low and high inertia configurations (×10^−3^ mm**)**

	Joint 1		Joint 2		Joint 3	
	High	Low	High	Low	High	Low
$r_{p}$	(0 185 673)^*T*^	(0–179 673)^*T*^	(-212 -319 673)^*T*^	(50 0 673)^*T*^	(212–319 673)^*T*^	(-50 0 673)^*T*^
Values	14.55	11.49	9.64	7.76	8.23	6.66

### Friction compensation approach

#### Stribeck model

In this section, we will briefly recall the formulation of Stribeck model^20^

(Equation 1) $$F\left(\dot{q}\right)=F_{c}\text{sgn}\left(\dot{q}\right)+\left(F_{s}-F_{c}\right)\exp\left({-\left(\dot{q}/{\dot{q}}_{s}\right)}^{2}\right)\text{sgn}\left(\dot{q}\right)+\sigma \dot{q}$$

where $F_{c}$ and $F_{s}$ represent the Coulomb frictional force and the maximum static force, $\dot{q}$ and ${\dot{q}}_{s}$ are the actuated joint velocity and the Stribeck velocity, and σ is the viscous coefficient. It can be seen from Equation 1 that the Stribeck model consists of three components, namely, Coulomb friction term, static friction term, and viscous friction term. The static friction part in the model is the same as the corresponding part in the LuGre model under steady state.^32^

It is worth pointing out that the Coulomb term and viscous term can be compensated by PID feedback controller and velocity feedforward controller respectively, while the static friction term can only be solved by designing a compensation algorithm for calculating its nonlinear characteristic. Furthermore, the change of inertia complicates the nonlinear characteristic, leading to the decrease in tracking accuracy of the robot.

#### The dynamic based approach

It is important to note that the amplitude of tracking error mutation caused by friction is affected by the inertia change, which is related to the configuration change of the TriMule robot as shown in Figure 5. This sudden change caused by static friction occurs at the starting and reversing of each actuated joint, which can be expressed as:

(Equation 2) $$F_{ss}=\left(F_{s}-F_{c}\right)\exp\left({-\left(\dot{q}/{\dot{q}}_{s}\right)}^{2}\right)\text{sgn}\left(\dot{q}\right)$$

Liu and Wu^33^ points out that the maximum static friction force $F_{s}$ and Coulomb friction force $F_{c}$ in a planar single-degree-of-freedom mechanical system have a linear relationship with their own equivalent mass, which can be expressed as:

(Equation 3) $$F_{s}-F_{c}=\left(\mu_{s}-\mu_{c}\right)M_{e}g$$

where $\mu_{s}$ and $\mu_{c}$ are the maximum static friction coefficient and the Coulomb friction coefficient, $M_{e}$ is the equivalent mass, and g is the gravitational acceleration. However, the parallel mechanism of the TriMule robot is a nonlinear time-varying system, leading to the friction varying with the inertia. Ignoring the weak coupling effect between the parallel mechanism and the wrist, the dynamic model of the parallel mechanism can be expressed as:^34^

$$F=M\left(q\right)\ddot{q}+H\left(q,\dot{q}\right)\dot{q}+G\left(q\right)$$

(Equation 4) $$\left[\begin{array}{l} F_{1}\\ F_{2}\\ F_{3} \end{array}\right]=\left[\begin{array}{ccc} M_{11} & M_{12} & M_{13}\\ M_{21} & M_{22} & M_{23}\\ M_{31} & M_{32} & M_{33} \end{array}\right]\left[\begin{array}{l} {\ddot{q}}_{1}\\ {\ddot{q}}_{2}\\ {\ddot{q}}_{3} \end{array}\right]+\left[\begin{array}{ccc} H_{11} & H_{12} & H_{13}\\ H_{21} & H_{22} & H_{23}\\ H_{31} & H_{32} & H_{33} \end{array}\right]\left[\begin{array}{l} {\dot{q}}_{1}\\ {\dot{q}}_{2}\\ {\dot{q}}_{3} \end{array}\right]+\left[\begin{array}{l} G_{1}\\ G_{2}\\ G_{3} \end{array}\right]$$

where $F\in R^{3\times 1}$ is the driving force vector, q, $\dot{q}$ and $\ddot{q}$ represent the position vector, velocity vector and acceleration vector respectively, $M\left(q\right)\in R^{3\times 3}$ is the inertia matrix, $H\left(q,\dot{q}\right)\in R^{3\times 1}$ is the Coriolis-centripetal matrix, and $G\left(q\right)\in R^{3\times 1}$ is the gravity vector. Accordingly, only considering the equivalent load inertia of the actuated joint itself, and without considering the coupling force between joints, velocity and gravity term, Equation 3 can be rewritten as:

(Equation 5) $$F_{s}-F_{c}=\left(\mu_{s}-\mu_{c}\right)M_{i}{\ddot{q}}_{i},i=1,2,3$$

where ${\ddot{q}}_{i}$ is the desired acceleration of the *i*-th actuated joint, and $M_{i}$ represents the *i*-th principal diagonal element of the inertia matrix M(q). Thus, the dynamic based friction model for each joint of the parallel mechanism can be expressed as:

(Equation 6) $$F_{s,i}=\left(\mu_{s}-\mu_{c}\right)M_{i}{\ddot{q}}_{i}\;\exp\left({-\left({\dot{q}}_{i}/{\dot{q}}_{s}\right)}^{2}\right)\text{sgn}\left({\dot{q}}_{i}\right)$$

#### The RBF based approach

During the starting and reversing phases, the inertial term in driving force plays a dominant role and the change in inertia affect the static friction significantly. Although the static friction model calculated by the dynamic can accurately reflect the relationship between the driving force dominated by the inertia term and the static friction force, higher computing power is required because of the complex dynamic of the parallel mechanism. Therefore, a novel approach for fitting the inertia variation in the friction model using RBF interpolation is proposed, which calculates the inertia value at any configuration in the workspace by performing spatial interpolation based on RBF on the inertia at a finite number of configurations. The inertia of the *i*-th actuated joint calculated by the RBF interpolation approach can be expressed as:

(Equation 7) $${\tilde{M}}_{i}=W_{i}^{T}\varphi ,i=1,2,3$$

where, $W_{i}={\left[w_{1,i},w_{2,i},\cdots ,w_{n,i}\right]}^{T}$ represents the weight vector of the *i*-th joint, $\varphi ={\left[\varphi_{1},\varphi_{2},\cdots ,\varphi_{n}\right]}^{T}$ is a Gaussian kernel function vector, and the *k*-th element of φ can be expressed as:

(Equation 8) $$\varphi_{k}=\exp\left(-{\left\|r_{p}-r_{p,k}\right\|}^{2}/2\zeta^{2}\right),k=1,2,..,n$$

where, $\left\|r_{p}-r_{p,k}\right\|$ denotes the distance between $r_{p}$ and $r_{p,k}$, $r_{p}$ denotes the position vector of the current *P*-point, $r_{p,k}$ denotes the position vector of the *k*-th sample point in the workspace, and *n* is the number of sample points, all the above vectors are measured in Κ−xyz. ζ is the width parameter of Gaussian kernel function.

$W_{i}$ can be calculated as:

(Equation 9) $$W_{i}=\Phi^{-1}M_{i}^{*},i=1,2,3$$

$$\left[\begin{array}{l} w_{1,i}\\ \vdots \\ w_{k,i}\\ \vdots \\ w_{n,i} \end{array}\right]={\left[\begin{array}{ccccc} \varphi_{1,1} & \cdots & \varphi_{1,k} & \cdots & \varphi_{1,n}\\ \vdots & \ddots & & & \vdots \\ \varphi_{m,1} & & \varphi_{m,k} & & \varphi_{m,n}\\ \vdots & & & \ddots & \vdots \\ \varphi_{n,1} & \cdots & \varphi_{n,k} & \cdots & \varphi_{n,n} \end{array}\right]}^{-1}\left[\begin{array}{l} M_{1,i}^{*}\\ \vdots \\ M_{m,i}^{*}\\ \vdots \\ M_{n,i}^{*} \end{array}\right]$$

$$\varphi_{m,k}=\exp\left(-{\left\|r_{p,m}-r_{p,k}\right\|}^{2}/2\zeta^{2}\right),k=1,2,..,n,m=1,2,..,n$$

where, $M_{i}^{*}={\left[M_{1,i}^{*},M_{2,i}^{*},\cdots ,M_{n,i}^{*}\right]}^{T}$ represents the known inertia vector composed of the inertia of the *i*-th joint at each sample point, Φ is a positive definite symmetric matrix with Gaussian kernel function, which is only related to the distance between the sample points. Accordingly, Equation 7 can be written as:

(Equation 10) $${\tilde{M}}_{i}=W_{i}^{T}\varphi ={\left(\Phi^{-1}M_{i}^{*}\right)}^{T}\varphi$$

In summary, the approximate inertia at any configuration in the workspace can be calculated by Equation 10. It is worth pointing out that $M_{i}^{*}$ and Φ are constant matrices that are calculated offline and stored in the controller, while only φ needs to be calculated online. Therefore, this approach avoids complex dynamic on-line operations and greatly reduces computational complexity while ensuring accuracy. The improved friction model using RBF interpolation method can be expressed as

(Equation 11) $$F_{f,i}=\left(\mu_{s}-\mu_{c}\right){\left(\Phi^{-1}M_{i}^{*}\right)}^{T}\varphi \;{\ddot{q}}_{i}\;\exp\left({-\left({\dot{q}}_{i}/{\dot{q}}_{s}\right)}^{2}\right)\text{sgn}\left({\dot{q}}_{i}\right)$$

## Results

Experiments to verify the effectiveness of the proposed simple and improved algorithms for friction compensation were carried out using the 3-DOF parallel mechanism within the TriMule robot developed for high-speed machining. The experiments were designed to examine two important issues: (1) the compensation ability for tracking errors in joint space at low and high inertia configurations; (2) the compensation ability for tracking errors in Cartesian space with 6-axis motion.

### Experiment configuration

**Figure 6:**
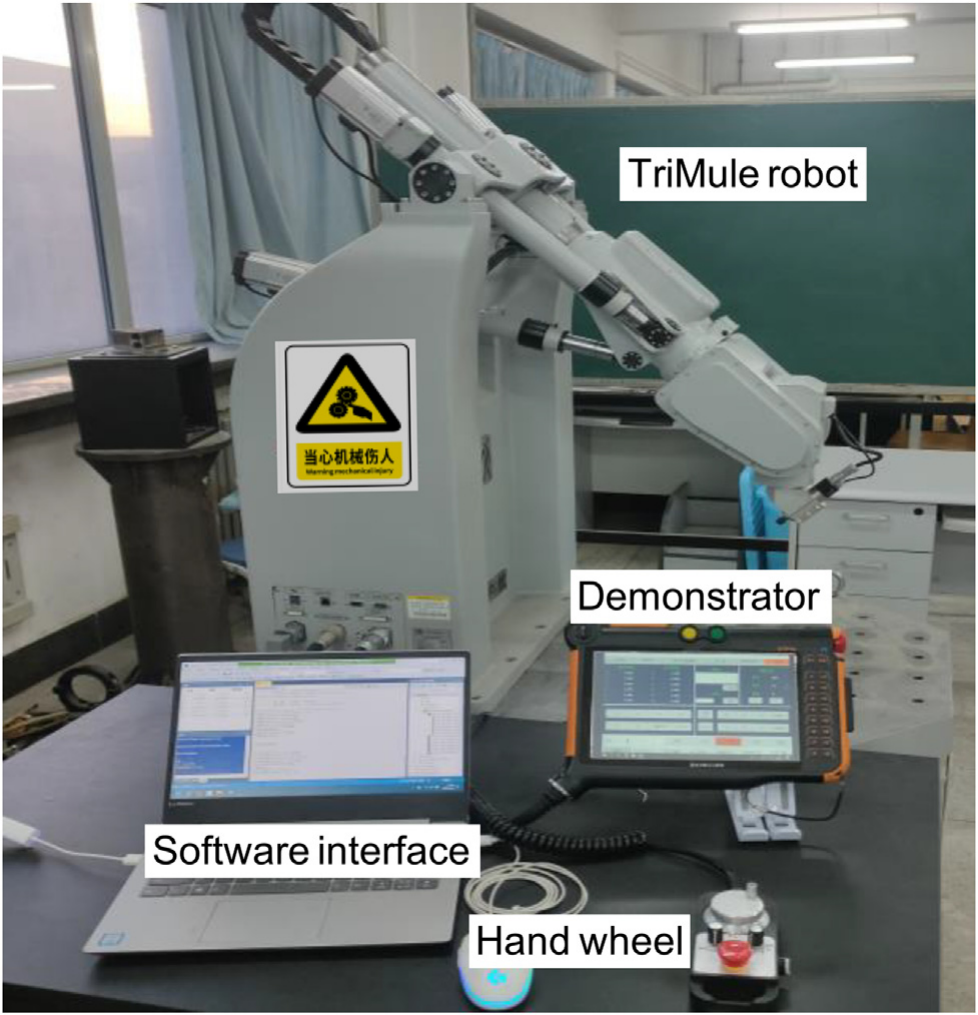
The TriMule robot

Figure 6 shows the prototype machine of the TriMule robot having the maximum movement capability of 400 mm/s in speed and 5 m/s^2^ in acceleration. The parallel mechanism is driven by three 400 W servo motors with ball screws, and the wrist is driven by three 200 W servo motors with cycloid planetary reducers. The control system includes a programmable multi-axis controller CK3M and servo drivers communicated over the EtherCAT network.

**Figure 7:**
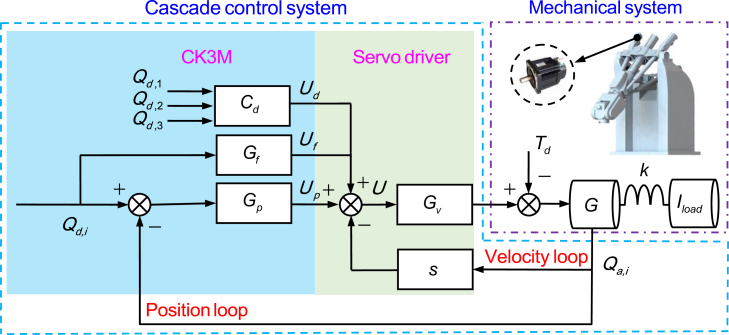
Control structure of each actuated joint

The control structure of each actuated joint is shown in Figure 7. $Q_{d,i}$ and $Q_{a,i}$ represent the desired and actual positions of the *i*-th actuated joint, $G_{p}$ and $G_{v}$ represents the position and velocity loop controllers respectively. $G_{f}$ represents the feedforward controller. $C_{d}$ represents the friction compensators designed based on the dynamic based model or the RBF based model, the dynamic program module or RBF interpolation module is stored in a high-brush buffer to calculate and modify the compensator parameters in real time. $T_{d}\left(s\right)$ is the actual friction disturbance, k is the joint stiffness, G is the controlled object (the motor), and $I_{load}$ represents the equivalent load inertia of the actuated joint. Taking the RBF based approach as an example, the outputs of friction compensator $C_{d}$, feedforward controller $G_{f}$, and position loop controller $G_{p}$ in the time domain can be represented as:

(Equation 12) $$u_{d}=\left(\mu_{s}-\mu_{c}\right){\left(\Phi^{-1}M_{i}^{*}\right)}^{T}\varphi \;{\ddot{q}}_{i}\;\exp\left({-\left({\dot{q}}_{i}/{\dot{q}}_{s}\right)}^{2}\right)\text{sgn}\left({\dot{q}}_{i}\right),i=1,2,3$$

(Equation 13) $$u_{f}=k_{v}{\dot{q}}_{i},i=1,2,3$$

(Equation 14) $$u_{p}=k_{p}e_{i}+k_{i}\int e_{i}dt+k_{d}{\dot{e}}_{i},i=1,2,3$$

Where, $k_{v}$ represents the feedforward gain, $e_{i}$ represents the tracking error of the *i*-th actuated joint, $k_{p}$, $k_{i}$ and $k_{d}$ represent the proportional, integral, and differential gain respectively. Then, the control voltage signal is

(Equation 15) $$u=u_{d}+u_{f}+u_{p}=\left(\mu_{s}-\mu_{c}\right){\left(\Phi^{-1}M_{i}^{*}\right)}^{T}\varphi \;{\ddot{q}}_{i}\;\exp\left({-\left({\dot{q}}_{i}/{\dot{q}}_{s}\right)}^{2}\right)\text{sgn}\left({\dot{q}}_{i}\right)+k_{v}{\dot{q}}_{i}+k_{p}e_{i}+k_{i}\int e_{i}dt+k_{d}{\dot{e}}_{i}$$

**Figure 8:**
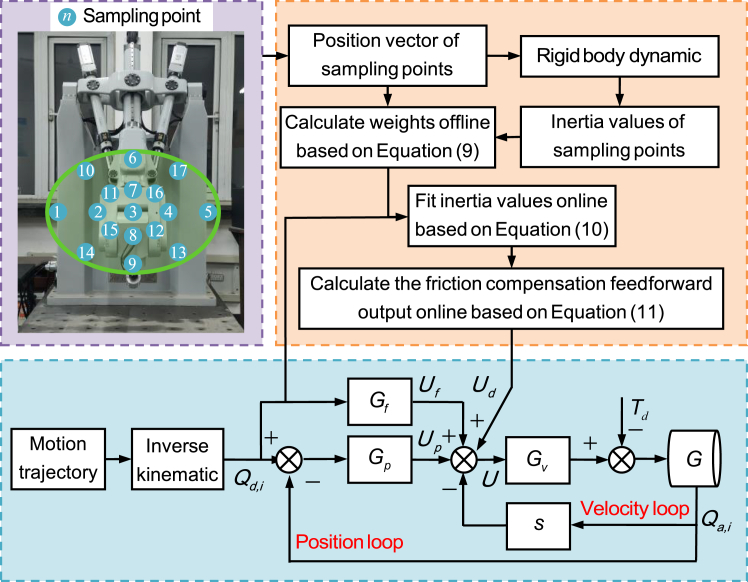
RBF-based friction compensation algorithm

Using the RBF approach to fit the inertia variation of the actuated joints in a parallel mechanism first requires collecting position vectors for multiple configurations and calculating the inertia of each actuated joint at the sampled configurations offline through dynamic computation. Subsequently, the weights are calculated offline using Equation 9 and stored in the controller. Then, these weights are used in Equation 10 to fit the inertia of the actuated joints online for the current configuration and in Equation 11 to compute the friction feedforward output based on the RBF approach. Figure 8 illustrates this process.

### Experiment in joint space

**Figure 9:**
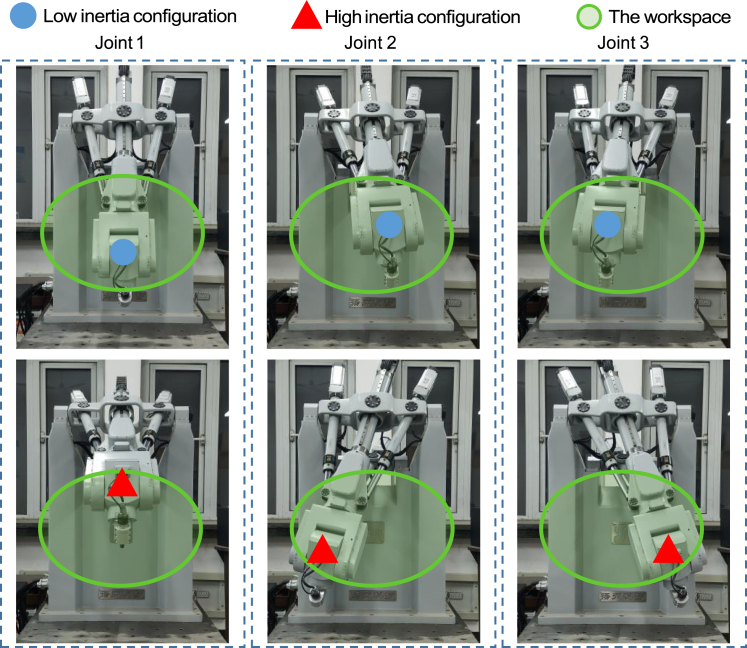
Low and high inertia configurations in the central plane (*x-y* plane) of the workspace

To verify the effectiveness of the compensation algorithm in adapting to inertia change, reciprocating motion experiments of each actuated joint of the parallel mechanism are carried out at the neighborhood of low and high inertia configurations as shown in Figure 9. The parabolic velocity signal is given for observing frictional disturbances conveniently, and the tracking errors are calculated by the CK3M motion controller with the actual positions collected by the motor encoders at a frequency of 2 kHz.

**Figure 10:**
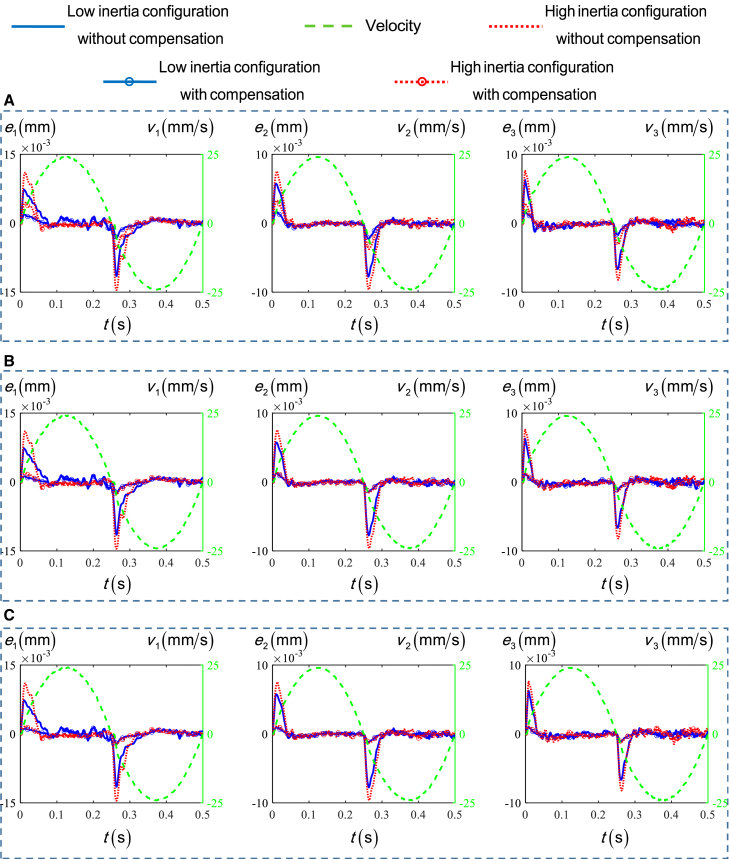
The compensation results of each compensation approach in the low and high inertia configurations of three actuated joints (A) Compensation results of TA for three parallel actuated joints. (B) Compensation results of RA for three parallel actuated joints. (C) Compensation results of DA for three parallel actuated joints.

Figure 10 shows the tracking errors of three actuate joints at different configurations with no compensation, the traditional approach using Stribeck model, the proposed RBF based approach, and dynamic based approach, denoted by NC, TA, RA, DA. The time histories are similar in general form, and their maximum values are listed in Table 2. It is worth noting that the tracking error is the difference between the actual position and the expected position measured by the encoder of each actuated joint motor, which is within the range of 0.01 mm after compensation. Since each actuated joint motor adopts a semi-closed-loop control, this tracking accuracy can be achieved by PID position feedback and velocity and acceleration feedforward control. When the position of each joint is located at its low inertia configuration, the maximum values of tracking errors of joints 1 drop by 71.80%, 78.59%, and 81.11% for TA, RA, and DA in comparison with NC. For joint 2 and joint 3, the maximum values of tracking errors are 71.01%, 81.96%, and 84.92% and 73.87%, 82.58%, and 84.08% lower for TA, RA, and DA in comparison with NC. Similar conclusions can be drawn at the high inertia configurations. In this case, the maximum values of tracking errors of joint 1 drop by 61.51%, 81.78%, and 83.89% for TA, RA, andDA in comparison with NC. For joint 2 and joint 3, the maximum values of tracking errors are 60.89%, 84.13%, and 86.62% and 63.67%, 84.08%, and 86.03% lower for TA, RA, and DA in comparison with NC.

**Table 2:** The maximum values of the tracking errors caused by friction in four cases at low and high inertia configurations of three actuated joints (×10^−3^ mm)

	Joint1		Joint 2		Joint 3	
	High	Low	High	Low	High	Low
NC	14.55	11.49	9.64	7.76	8.23	6.66
TA	5.60	3.24	3.77	2.25	2.99	1.74
SA	2.66	2.46	1.53	1.40	1.31	1.16
CA	2.31	2.17	1.29	1.17	1.15	1.06

It can be seen that the compensation effect of TA at the high inertia configuration is about 10% lower than that at the low inertia configuration, due to the friction model parameters are identified at the neighborhood of the low inertia configurations which are not applicable to the high inertia configurations. Compared to the case with TA, the tracking accuracy of three joints can be further increased by up to 10.95%, 13.91% at low inertia configurations and by up to 23.24%, 25.73% at high inertia configurations for RA and DA. This confirms the argument that considering the inertia change helps significantly to improve the joint tracking accuracy, especially when the parallel mechanism operates at different configurations.

### Experiment in cartesian space

**Figure 11:**
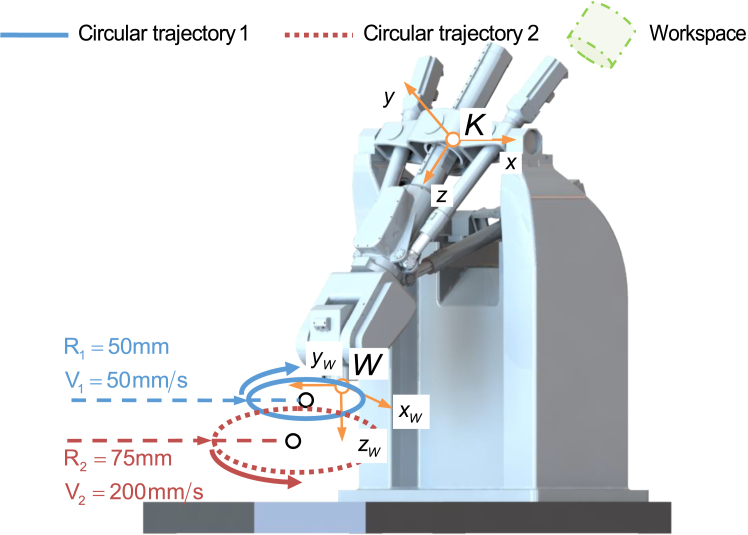
The verification trajectories with two different configurations in the workspace

To verify the effectiveness of the proposed algorithms, two circular trajectories with different configurations as shown in Figure 11 are planned in the workspace aiming to prove the improvement of tracking accuracy in Cartesian space. The circular trajectory 1 and the circular trajectory 2 are planned in the *x*_*W*_-*y*_*W*_ plane of the *W* coordinate system. The circular trajectory 1 is located in the center area of the workbench, and the circular trajectory 2 is below the circular trajectory 1 and its boundary is close to the reachable workspace boundary of the workbench. The configurations of the two circular trajectories are listed in the Table 3. During the motion, the actuated joints of the parallel mechanism experience at least one motor reversal to reflect the impact of friction disturbances on the tracking errors. Here, the tracking errors caused by friction in parallel mechanism are compensated by using TA, RA, and DA, while the tracking errors in the wrist are only compensated by using TA due to the approximate invariance of inertia.

**Table 3:** The configurations of the two circular trajectories

	Direction	Radius (mm)	Center position (mm)	Velocity (mm/s)
Circular trajectory 1	Clockwise	50	(0, 50, 0)^T^	50
Circular trajectory 2	Counterclockwise	75	(0, 45, 30)^T^	200

**Figure 12:**
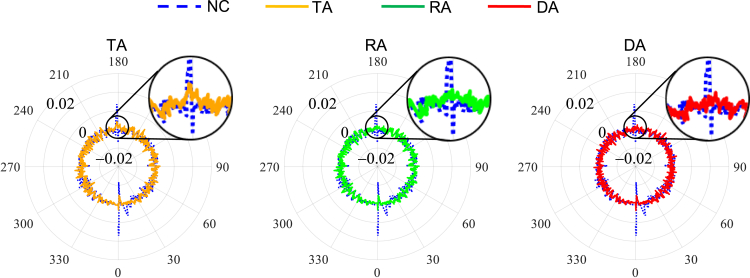
The polar coordinate diagram of the tracking errors on the circular trajectory 1 after compensation by using three approaches

Figures 12 and S3 show the polar coordinate diagram of the tracking errors on the circular trajectory 1 and the circular trajectory 2 after compensation by using three approaches. The maximum values of the tracking errors on the circular trajectory 1 and the circular trajectory 2 are shown in Table 4. It is worth pointing out that the tracking errors in Figures 12 and S3 are obtained by each actuated joint tracking error through the forward kinematic algorithm, so as to show the influence of the actuated joint friction on the tracking accuracy of the terminal trajectory. The tracking errors only considers the result of mapping the tracking error of the joint motor motion to the terminal and does not include the robot assembly error, as well as the motion transmission error and mechanism deformation error after the motor shaft output end, etc. Therefore, the tracking error is less than the end positioning error or contour error during the actual motion process of the robot. Compared with the case with NC, the peak of errors on the circular trajectory 1 and the circular trajectory 2 are reduced by 69.55%, 81.37%, and 83.89% and 71.76%, 83.29%, and 85.85%, respectively, after compensation by using TA, RA, and DA. Moreover, the compensation effect of DA is less than 3% higher than that of RA, which verifies that the RBF based algorithm and the dynamic based algorithm have an approximate effect.

**Table 4:** The maximum values of tracking errors caused by friction in four cases (×10^−3^ mm)

	NC	TA	RA	DA
Circular trajectory 1	13.53	4.12	2.52	2.18
Circular trajectory 2	16.04	4.53	2.68	2.27

**Figure 13:**
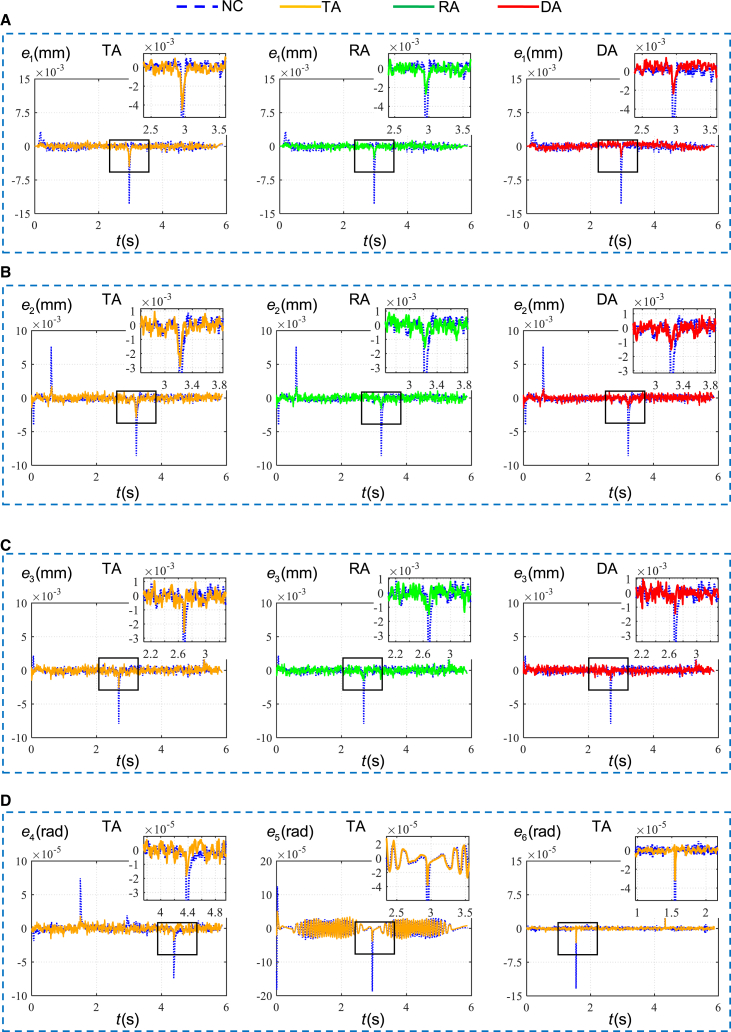
The tracking errors of the actuated joint 1–6 for the robot moving along the circular trajectory 1 (A) Compensation results of joint 1 by three approaches. (B) Compensation results of joint 2 by three approaches. (C) Compensation results of joint 3 by three approaches. (D) Compensation results of the wrist joints by TA.

Figures 13 and S4 show the tracking errors of the actuated joint 1–6 for the robot moving along the circular trajectory 1 and the circular trajectory 2, and the maximum values of that are listed in Table 5. Compared to the case with TA, the maximum values of tracking errors of the actuated joint 1–6 for the robot moving along the circular trajectory 1 and the circular trajectory 2 are reduced by more than 15.1% and 20.84% for RA and by more than 16.48% and 23.07% for DA. The circular trajectories with different configurations fully reflect the influence of inertia change on friction disturbance. The low amplitudes of these tracking errors confirm the great compensation capability of the proposed approach and its extended applicability to position and velocity.

**Table 5:** The maximum values of the tracking errors of the actuated parallel joint 1–3 (×10^−3^ mm) and the actuated series joint 4–6 (×10^−4^ rad)

	NC		TA		RA		DA	
	1^a^	2^b^	1	2	1	2	1	2
Joint 1	12.87	15.36	4.49	5.97	2.68	2.77	2.37	2.40
Joint 2	8.61	11.31	2.88	4.30	1.58	1.87	1.52	1.55
Joint 3	7.96	10.84	2.57	3.98	1.51	1.69	1.46	1.48
Joint 4	0.74	0.73	0.18	0.19				
Joint 5	1.87	2.05	0.40	0.46				
Joint 6	1.34	1.29	0.32	0.30				

a Circular trajectory 1.

b Circular trajectory 2.

## Discussion

### Conclusions

This paper explores an improved friction compensation approach considering inertia change. The conclusions are drawn as follows.

(1) The contribution proportion of the friction effect between the parallel mechanism and the wrist is revealed, and the inertia variation across the whole workspace is analyzed. The tracking errors of each actuated joint of the parallel mechanism can be increased by up to 21% at the neighborhood of high inertia configurations compared with the case at low inertia configurations, leading to the non-ignorable influence of the inertia change on friction is discovered.
(2) A friction compensation approach considering the inertia change is proposed by introducing the inertia term of the joint driving force into the traditional Stribeck model as thrust to eliminate the mutation phenomenon of the errors occurring at the starting and reversing stage caused by static friction. On this basis, the inertia change in the model is fitted by radial basis function interpolation, which reduces the calculation cost while ensuring the accuracy.
(3) Experimental results on the parallel mechanism within a 6-axis hybrid robot named TriMule show that the proposed algorithm improves the tracking accuracy significantly and provides good extrapolation capability for different configurations. The results also show that the acceptable tracking errors can be ensured by the RBF based approach, which is similar to that with the dynamic based approach. Considering the calculation efficiency and cost of the controller, the RBF based approach achieves satisfactory compensation results and has better cost-effectiveness.

### Limitations of the study

In the future work, the different friction characteristics in different directions caused by the gravity term will be considered in the model. In addition, a new encoder needs to be installed at the end of the actuator so that the robot can achieve full closed-loop control to consider the issues such as joint backlash caused by assembly errors, thereby further improving the accuracy of the terminal.

## Resource availability

### Lead contact

Further information and requests for resources and reagents should be directed to and will be fulfilled by the lead contact, Bin Li (E-mail: cnrobot@tjut.edu.cn).

### Materials availability

This study did not generate new materials.

### Data and code availability

• All data reported in this paper will be shared by the lead contact upon reasonable request.
• All code in this paper will be shared by the lead contact upon reasonable request.
• Any additional information required to reanalyze the data reported in this paper is available from the lead contact upon reasonable request.

## Acknowledgments

This work was supported by the 10.13039/501100001809National Natural Science Foundation of China (NSFC) (grant 52205029, 52205030), the 10.13039/501100006606Natural Science Foundation of Tianjin (23JCQNJC00430), the 10.13039/501100011381State Key Laboratory of Robotics and Systems (HIT) (SKLRS-2023-KF-07).

## Author contributions

Q.L., conceptualization, funding acquisition, writing – original draft, writing – review and editing; S.S., data curation, formal analysis, investigation, methodology, validation, visualization, writing – original draft, and writing – review and editing; Y.M., funding acquisition, methodology, and resources; B.L., funding acquisition, methodology, writing – original draft, and writing – review and editing.

## Declaration of interests

The authors declare no competing interests.

## STAR★Methods

### Key resources table

**Table:** 

REAGENT or RESOURCE	SOURCE	IDENTIFIER
**Software and algorithms**		

MATLAB	Mathworks, Inc.	N/A
SOLIDWORKS	Dassault Systemes SOLIDWORKS Corp	N/A

### Method details

#### The weight parameters of the RBF approach

The weight parameters of the RBF approach depend on the positions of the sampling points and the inertia values of the sampled configurations calculated based on dynamics. Figure 8 also illustrates all sampling points selected on the central plane of the workspace (*x-y* plane). The sampling points are evenly distributed along the gradient from the minimum to the maximum inertia values of each drive joint on the current plane. A total of 9 sampling points are selected for each drive joint in the *x-y* plane, and the weights calculated using the RBF spatial interpolation method are listed in Table S1.

#### The fitting effect of RBF method on inertia values

It can be observed from Figure 5 that the inertia of each actuated joint changes relatively smoothly with the configuration in the global workspace. Therefore, under the condition that the maximum fitting errors of the load inertia for each joint across the entire workspace is less than 5 $\text{kg}\cdot mm^{2}$, width parameters ζ of 0.6, 0.5, and 0.5 are selected for joint 1, 2, and 3 of the parallel mechanism, respectively. Figure S1 illustrates the errors between the inertia values calculated by using rigid body dynamics and those fitted by using the RBF approach on the central plane of the workspace (*x-y* plane). It can be observed that the error for joint 1 is less than 1.23 $\text{kg}\cdot mm^{2}$, while the errors for joints 2 and 3 are less than 3.91 $\text{kg}\cdot mm^{2}$. The errors of all the actuated joints are within 2.48% of the minimum inertia value. The above results indicate that the RBF approach can effectively fit the inertia value across the entire workspace of each actuated joint. Furthermore, Figure S2 illustrates the variation of friction force with velocity and inertia values in proposed model.

#### Friction parameter identification

This paper identifies the friction model parameters offline using the nonlinear least squares method. First, the motor actuated signals of the actuated joints at different velocity levels in the center of the workspace are collected. By using the dynamic characteristics, the variation of friction force with respect to motor shaft velocity is obtained. Then, the friction curve is fitted using the nonlinear least squares method, and the friction model parameters are identified accordingly.^35^ The identified model parameters are listed in the Table S2.

### Quantification and statistical analysis

All statistical analyses and results are described in the relevant sections. The raw experimental data in the part of RESULTS was measured by the encoders of the motor and collected by POWER PMAC IDE with a sampling frequency of 2kHz. Figures were produced by MATLAB R2020a from the raw data.
