# Supplementary material for: A friction compensation approach of a 6 axis hybrid robot by considering joint inertia change
Source: iScience. 2025 Mar 6;28(4):112173. doi: 10.1016/j.isci.2025.112173 (PMC11987701; doi:10.1016/j.isci.2025.112173)
Supplement: Document S1. Figures S1–S4 and Tables S1 and S2 [file mmc1.pdf]

iScience, Volume *28*

Supplemental information

A friction compensation approach of a 6 axis hybrid robot by considering joint inertia change

Qi Liu, Sitong Shen, Yue Ma, and Bin Li

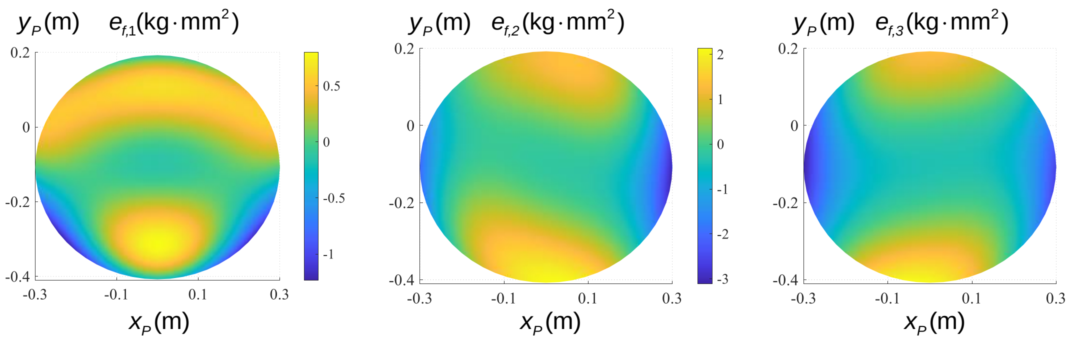

Figure S1. The fitting error of inertia values for each actuated joint (*x*-*y* plane)

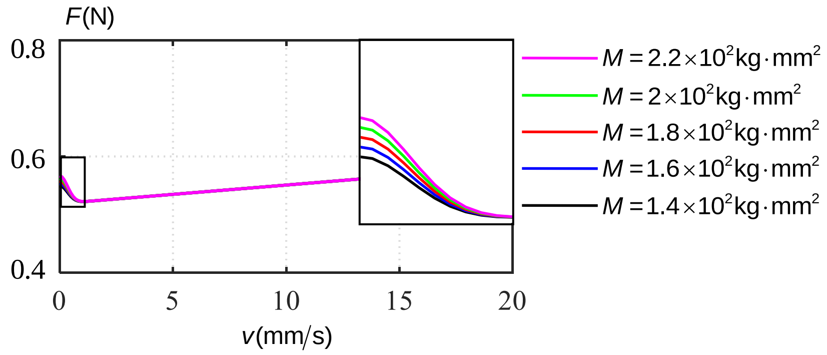

Figure S2. The variation of friction force with velocity and inertia values

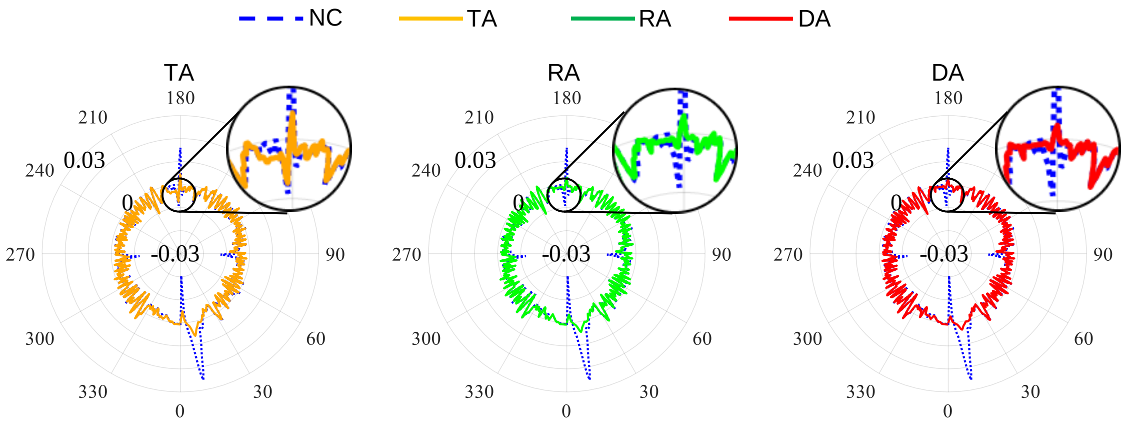

Figure S3. The polar coordinate diagram of the tracking errors on the circular trajectory 2 after compensation by using three approaches

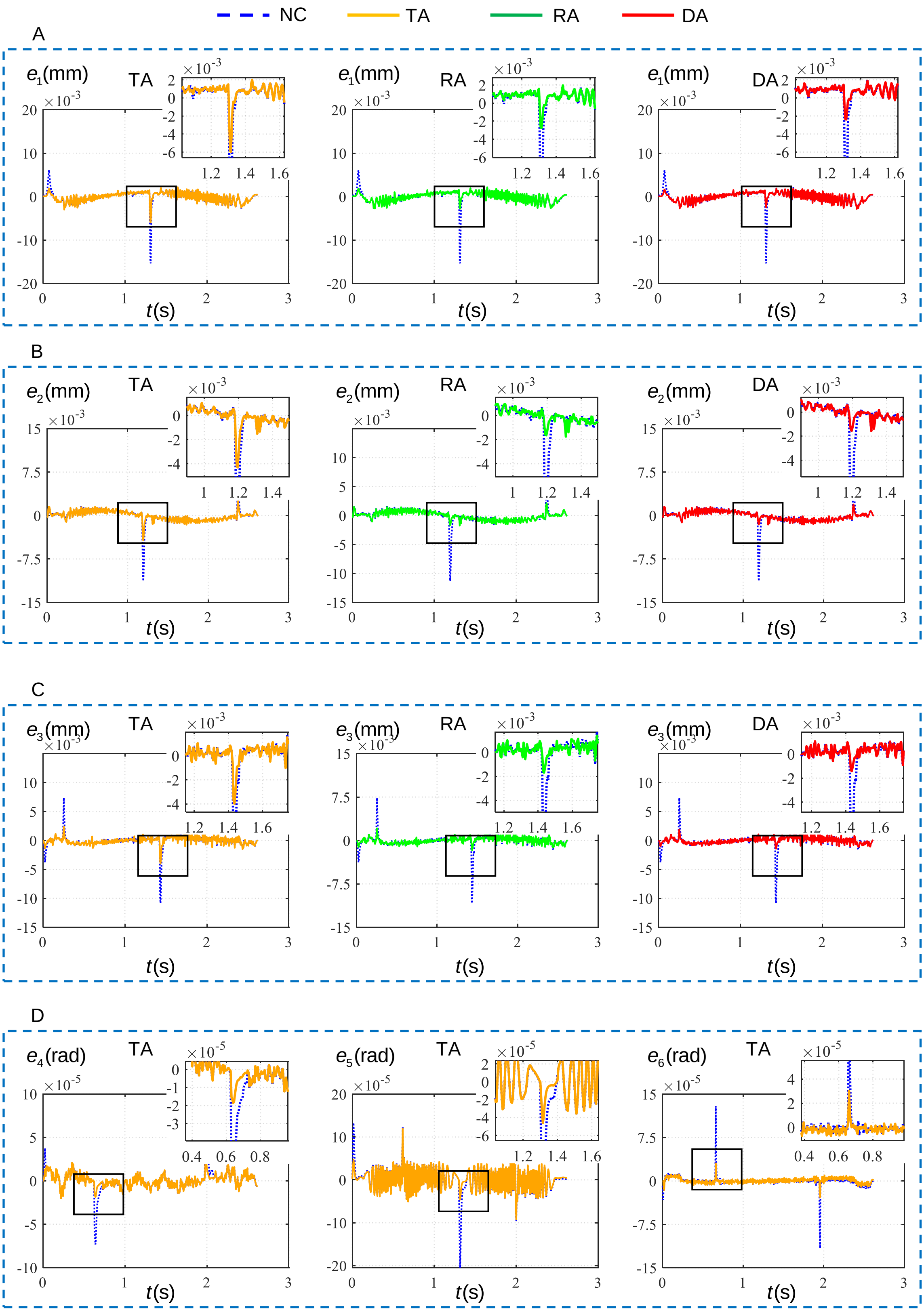

Figure S4. The tracking errors of the actuated joint 1~6 for the robot moving along the circular trajectory 2

(A) Compensation results of joint 1 by three approaches.

(B) Compensation results of joint 2 by three approaches.

(C) Compensation results of joint 3 by three approaches.

(D) Compensation results of the wrist joints by TA.

Table S1 The weights of sampling points for each actuated joint

Joint 1									
Sampling points	1	2	3	4	5	6	7	8	9
Weights	0.0134	0.0135	0.1287	0.0101	0.0047	0.1246	-0.4247	-0.6168	0.2294
Joint 2									
Sampling points	10	11	3	12	13	14	15	16	17
Weights	0.0163	-0.0331	0.1801	-0.0486	0.0250	0.2058	-0.6357	-0.3021	0.0934
Joint 3									
Sampling points	10	11	3	12	13	14	15	16	17
Weights	0.0883	-0.2854	0.1792	-0.6030	0.1961	0.0358	-0.0816	-0.0603	0.0237

Table S2 Identification results of friction parameters for each actuated joint

	Joint 1	Joint 2	Joint 3	Joint 4	Joint 5	Joint 6
F_c(N)	0.5188	0.5032	0.4983	0.3488	0.3721	0.3452
F_s(N)	0.5536	0.5262	0.5168	0.3676	0.3948	0.3624
$\dot{q}_s$(mm/s)	0.4842	0.6641	0.7543	1.1647	0.8844	1.2540
σ(N·s/mm)	0.80×10^{-3}	0.63×10^{-3}	0.69×10^{-3}	0.35×10^{-3}	0.5×10^{-3}	0.41×10^{-3}
